# ATR prevents Ca^2+^ overload-induced necrotic cell death through phosphorylation-mediated inactivation of PARP1 without DNA damage signaling

**DOI:** 10.1096/fj.202001636RRR

**Published:** 2021-05

**Authors:** Zhengke Li, Hui Wang-Heaton, Brian M. Cartwright, Yetunde Makinwa, Benjamin A. Hilton, Phillip R. Musich, Nikolozi Shkriabai, Mamuka Kvaratskhelia, Shengheng Guan, Qian Chen, Xiaochun Yu, Yue Zou

**Affiliations:** 1 Department of Biomedical Sciences, Quillen College of Medicine, East Tennessee State University, Johnson City, TN, USA; 2 Department of Cancer Biology, University of Toledo College of Medicine, Toledo, OH, USA; 3 Department of Medicine, University of Colorado School of Medicine, Aurora, CO, USA; 4 Department of Pharmaceutical Chemistry and Mass Spectrometry Facility, University of California, San Francisco, CA, USA; 5 Department of Cancer Genetics and Epigenetics, City of Hope, Duarte, CA, USA

**Keywords:** ATR, Ca^2+^ overload, necrosis, PARP1, PARP1 phosphorylation

## Abstract

Hyperactivation of PARP1 is known to be a major cause of necrotic cell death by depleting NAD^+^/ATP pools during Ca^2+^ overload which is associated with many ischemic diseases. However, little is known about how PARP1 hyperactivity is regulated during calcium overload. In this study we show that ATR kinase, well known for its role in DNA damage responses, suppresses ionomycin, glutamate, or quinolinic acid-induced necrotic death of cells including SH-SY5Y neuronal cells. We found that the inhibition of necrosis requires the kinase activity of ATR. Specifically, ATR binds to and phosphorylates PARP1 at Ser179 after the ionophore treatments. This site-specific phosphorylation inactivates PARP1, inhibiting ionophore-induced necrosis. Strikingly, all of this occurs in the absence of detectable DNA damage and signaling up to 8 hours after ionophore treatment. Furthermore, little AIF was released from mitochondria/cytoplasm for nuclear import, supporting the necrotic type of cell death in the early period of the treatments. Our results reveal a novel ATR-mediated anti-necrotic mechanism in the cellular stress response to calcium influx without DNA damage signaling.

## 1 | INTRODUCTION

An intracellular spike of Ca^2+^ is cytotoxic and a major cause of ischemic injuries.^1–8^ Ca^2+^ overload plays a critical role in the pathogenesis of ischemic diseases by inducing cell death via necrosis, apoptosis, or parthanatos.^2,3,9–22^ Necrosis is a major type of ischemic cell death and occurs early after ischemic injuries.^2–5,9–17,21–27^ Thus, necrosis induced by a calcium spike contributes to diseases such as heart failure, ischemic stroke, brain injury, and immune diseases.^2,6–8^ Despite these, the molecular mechanisms of Ca^2+^ overload-induced necrotic cell death remain elusive.

Necrotic cell death was traditionally thought to be an uncontrolled process resulting from cellular infection or physicochemical stress. Necrosis is characterized by cytoplasmic swelling, irreversible and rapid loss of plasma membrane integrity, and spillage of the intracellular components.^28–30^ Recently, there has been a growing body of evidence demonstrating that necrotic cell death also is a regulated process and plays an important role in maintaining the physiologic health of cell populations.^28,31^ For example, necrosis may serve to maintain tissue and organism integrity by initiating inflammatory responses: high mobility group box 1 (HMGB1) protein, as a nuclear protein that is released from necrotic cells, acts as a signaling molecule to inform neighboring cells that tissue repair is required.^32–35^ How necrosis is regulated intracellularly and how cells respond to the Ca^2+^ overload stress remains largely elusive, particularly in terms of the mechanism of the induced necrosis. Hyper-activation of poly(ADP-ribose) polymerase 1 (PARP1) toward poly-(ADP-ribosyl) ation (PARylation) has been demonstrated to be a major cause of necrotic cell death by depleting the NAD^+^ pool and, thus, the NAD^+^ supply for ATP production in mitochondrial, leading to ATP depletion.^36–39^ Protein PARylaton is required for many cellular physiological and pathological processes, including DNA repair and cell death.^40–44^ PARP1 is a highly active enzyme and is responsible for producing up to 90% of the poly (ADP-ribose) (PAR) polymers within cells.^45–47^ However, hyperactivation of PARP1 leads to excessive consumption of cellular NAD^+^, which results in blockage of glycolytic activity followed by a fall in ATP levels and the accumulation of phosphorylated sugars.^48–53^ The depletion of the NAD^+^/ATP pools leads to necrosis due to metabolic and bioenergetic collapse.^54^ In addition, upon Ca^2+^ influx into cells, mitochondrial activity is decreased, facilitated by further Ca^2+^ release from the endoplasmic reticulum (ER), leading to increased levels of reactive oxygen species (ROS) and reduced levels of ATP, which results in the induction of necrotic cell death.^33,55^

Ataxia telangiectasia and Rad3-related (ATR) protein is a phosphoinositide 3-kinase-related protein kinase and is well known for its regulatory role in DNA damage responses (DDR). It is a critical kinase for maintaining genome integrity through its roles in regulating DNA replication and cellular defense responses to DNA damage caused by endogenous or environmental sources.^56–59^ In response to DNA damage, ATR and the related kinase *ataxia telangiectasia* mutated (ATM) activate multiple checkpoint-signaling pathways to arrest the cell cycle, promote DNA repair, and/or apoptosis to eliminate the damaged cells.^60^ ATR is activated by DNA damage-induced replicative stress, while ATM primarily is activated by DNA double-strand breaks (DSBs).^61,62^ When activated, ATR phosphorylates hundreds of downstream protein substrates such as checkpoint kinase 1 (Chk1) and the tumor suppressor p53,^63–65^ which are called the mediators or effectors that carry out specific critical roles in the stress response. ATR phosphorylates substrates at sites with serine or threonine residues that are followed by a glutamine residue (the SQ/TQ motif).^66–68^ To our knowledge, there have been no reported observations that ATR plays a role in programmed necrosis.

This study explores the role of ATR kinase in regulating Ca^2+^ overload-induced necrosis in the absence of DNA damage-signaling activation. We demonstrate that the kinase activity of ATR is required to suppress Ca^2+^overload-induced necrosis independent of ATR-mediated DNA damage signaling. PARP1-mediated PARylation is the target of ATR’s role in moderating the Ca^2+^ overload-induced necrosis. Mechanistically, ATR phosphorylates PARP1 at serine 179, which suppresses PARylation and, thus, Ca^2+^ overload-induced necrosis. Our study identifies the ATR protein kinase as an important new player in regulating necrosis, and our results highlight a potential application of ATR kinase inhibitors in moderating necrosis-related diseases.

## 2 | MATERIALS AND METHODS

### 2.1 | Cell culture, reagents, and antibodies

Cells were cultured in Dulbecco’s modified Eagle’s medium (DMEM) supplemented with 10% FBS and 1% penicillin/streptomycin. The ATR inhibitor VE-822 (cat# B1383) was from ApexBio Technology. Antibodies against phospho-Chk1 (S345; cat# 2348), Chk1 (cat# 2360), actin (Cat# 4970), PARP1 (cat# 9542), cleaved caspases 3 (cat# 9661) and 7 (cat# 9491), γ-H2AX (A300–081A), ATM (cat# A300–299A), and GAPDH (cat# 2118) were from Cell Signaling Technology. The antibody against ATR (cat# A300–137A) was from Bethyl Laboratories. The phospho-ATR antibody (T1989; cat# GTX128145) and HMGB1 antibody (cat# GTX101277) were from GeneTex. Antibody against poly (ADP-ribose) (PAR) (Cat#4338-MC-50) was from Trevigin.

### 2.2 | Western blotting

Whole cell lysates were prepared by incubating harvested cells in lysis buffer [50 mM Tris-HCl pH 8.0, 140 mM NaCl, 1% Triton X-100, 0.05% SDS, 1 mM EDTA, and 1× protease and phosphatase inhibitors (Roche)] on ice for 10 minutes, and then centrifuged at 20 000 *g* for 10 minutes at 4°C to clear the lysates. After the addition of an equal volume of 2 × SDS loading buffer the lysates were boiled for 10 minutes before separation by SDS-PAGE for western blot (WB) analysis. After electroblotting onto PVDF membranes specific proteins were detected using the antibodies specified above. In most cases, the blots were probed sequentially with different antibodies without stripping if the probed proteins have very different molecular weights and migrated as well-separated bands on SDS-PAGE. However, there were several cases that the probed proteins had close migrations on the gels so that blots were stripped for separate probing.

### 2.3 | Immunofluorescence microscopy

For immunofluorescence detection of PARP1 protein and PAR-modified proteins, cells were grown on coverslips before the initiation of experimental treatments. After treatment, cells were fixed with 4% paraformaldehyde, permeablized with 0.25% Triton X-100 in PBS and blocked with 5% bovine serum albumin (BSA) for 1 hour at room temperature. Phosphorylated proteins were detected with the indicated antibodies, and appropriate fluorescence-conjugated secondary antibodies (Thermo Fisher Scientific). The cells on coverslips were mounted with ProLong Gold anti-fade reagent containing DAPI (Thermo Fisher Scientific) before microscopy.

### 2.4 | Duolink proximity ligation assays

For Duolink microscopic detection of protein interactions cells were grown on coverslips before the initiation of experimental treatments. After ionomycin treatment, cells were fixed with 4% paraformaldehyde and permeablized with 0.2% Triton X-100 in PBS and blocked with 3% BSA in PBS for 1 hour at room temperature. Proteins then were detected with primary antibodies and the proximity ligation assay (PLA) assay was carried out according to the company’s protocol (Sigma, DUO92101). Images were taken using the Life Technologies Evos FL Auto microscope or an Olympus confocal microscope.

### 2.5 | Subcellular fractionation

Subcellular fractionation was carried out as described previously.^69,70^ Briefly, cytoplasmic and nuclear extracts were obtained following the subcellular fractionation protocol of the ProteoJETTM cytoplasmic and nuclear protein extraction kit (ThermoFisher). Briefly, 10 volumes of cell lysis buffer (with 1x protease inhibitors) were added to 1 volume of packed cells. After a short vortexing and incubation on ice for 10 minutes, cytoplasm was separated from nuclei by centrifugation at 500 *g* for 7 minutes at 4°C. Isolated nuclei were washed with 500 uL of the nuclear washing buffer and collected by centrifugation. The washed nuclei were lysed in ice-cold buffer (140 mM NaCl, 50mM Tris-HCl pH7.8, 10% glycerol, 1% NP-40, and 1 mM EDTA, with 1X protease and phosphatase inhibitors added freshly before use) in a 1.5 mL microcentrifuge tube rotated at 4°C for 15 minutes. Nuclear lysate was the supernatant after centrifugation at 13 000 rpm for 10 minutes at 4°C. Then equal volume of 2 × SDS loading buffer was added to each fraction for western blotting. In all of the fractionation experiments western blotting of either cytoplasmic (β-actin or GAPDH) and nuclear (PARP) markers were assessed to check successful fractionation and cytoplasmic and nuclear protein loading, respectively.

### 2.6 | Immunoprecipitation

Cytoplasmic and nuclear extracts were collected as described above.^69,70^ The extracts were diluted to 1 mL with ice-cold buffer (50 mM Tris-HCl pH 7.8, 140 mM NaCl, 10% glycerol, 1% NP-40, 1 mM EDTA, plus 1x protease, and phosphatase inhibitor cocktail from Thermo Fisher Scientific). For each 1 mg of protein, 1 μL of anti-PARP1 antibody was added. The mix was incubated overnight at 4°C on a rotator. About 50 μL of Protein G agarose slurry was added to conjugate the antigen-antibody complex for 4 hours on rotation at 4°C. Bead-bound proteins were collected by centrifugation and washed three times with washing buffer (50 mM Tris-HCl pH 7.6, 140 mM NaCl, 10% glycerol, 0.2% Tween 20, 1 mM EDTA supplied with proteinase and phosphatase inhibitors). Proteins on beads were eluted by adding 2 × SDS loading buffer and boiling for 10 minutes.

### 2.7 | Exogenous protein expression

For transient transfections, specified cDNA expression plasmids were diluted into jetPEI DNA transfection reagent; transfection was done by following the PolyPlus protocol for adherent cells. Briefly, cells were seeded at 30%−50% confluency 1 day before transfection which was continued for 48 hours.

### 2.8 | Isolation of cell clones with stable protein-expressing constructs

Antibiotic selection was employed 48 hours after transfection to select cells containing a plasmid construct and to stabilize the expression of the exogenous protein construct. The antibiotic selection was maintained for 14 days with replacement of the selective medium every 3 or 4 days. Cells were harvested and diluted to 1 cell per 0.5 mL culture medium and plated at 0.3 mL per well into 96-well plates. Large and healthy colonies (500–1000 cells) were isolated using sterile 10 μL micropipette tips and expanded in culture medium containing the appropriate antibiotic. WB analysis was performed to confirm that cells continue to express the desired protein.

### 2.9 | Trypan blue exclusion assay

Dead cells with a fractured cell membrane take up the dye trypan blue. Thus, a trypan blue exclusion assay was employed to determine necrotic cell death. About 0.1 mL of a 0.4% trypan blue stock solution was added to a 1 mL of suspension of cells that were harvested from both attached and floating cells. The number of blue cells were counted against total number of cells; the percentage of necrotic cells was calculated as (# blue cells/# total cells) × 100.

### 2.10 | Cell titer-blue cell viability assay

The procedure was done by following the protocol of the Cell Titer-Blue Reagent from Promega. Briefly, cells were seeded in wells of a 96-well microplate. Cell treatments occurred the next day, after which Cell Titer-Blue reagent was added at 5 μL per well. The plate was incubated at 37°C for another 2 hours. The fluorescence [560Ex/590Em] was recorded using a fluorescent microplate reader. This assay is based on the ability of living cells to convert the cell-permeable redox dye resazurin to resorufin, which fluoresces at 590 nm. A decrease in fluorescence intensity reflects a decrease in cell viability.

### 2.11 | LDH release assay

The assay measures the lactate dehydrogenase (LDH) released by dying cells into the culture supernatant. The released LDH converts a tetrazolium salt (INT) into a red formazan product. The amount of color formed is proportional to the number of lysed/dead cells. Briefly, cells were seeded in wells of a 96-well microplate 1 day ahead of the cell treatment and assay. The CytoTox 96 Reagent Kit was employed (Promega cat#G1780). Following experimental treatment, 50 μL of media from each well are transferred to another 96-well plate. Several non-treated wells of the culture plate received a 1:10 dilution of Lysis Solution before the media transfer and incubated for 45 minutes to lyse all cells as a positive control. For the LDL assay 50 μL of CytoTox 96 Reagent is added to each well containing 50 μL of transferred media and the assay plate was incubated for 30 minutes at room temperature, followed by the addition of 50 μL of Stop Solution. The assay plate was incubated for another 15 minutes at room temperature. The absorbance signal at 490 nM was measured in a microplate reader.

#### 2.11.1 | Propidium iodide (PI) staining

PI was employed in some experiments to determine the loss of cell membrane integrity as it enters cells only if the plasma membrane becomes permeable and stains DNA and RNA. Hoechst 33343 is a cell membrane-permeable nuclear counterstain that emits blue fluorescence when bound to dsDNA. Both dyes were added simultaneously to determine the ionomycin-induced necrotic cell death. Briefly, after ionomycin treatment, cells were washed with 37°C-warmed 1 × PBS. PI and Hoechst 33342 were diluted into 37°C-warmed 1 × PBS at 5 μg/mL and added to the attached cells and incubated for 10 minutes at 37°C. The staining mix was then discarded, and cells were washed with 37°C-warmed 1 × PBS. Images were captured with an EVOS fluorescent microscope and PI-stained cells were quantified relative to the total cell number (stained with Hoechst 33342).

### 2.12 | Intracellular calcium concentration measurement

HCT116 ATR^+/+^ and ATR^flox/−^ (ATR-proficient and -deficient) cells were grown on glass coverslips and then loaded with Fura2 for 30 minutes at room temperature. Cells were washed with PBS to remove excess Fura2 and then transferred to an acrylic chamber on the microscope stage. Fluorescence measurements were recorded as soon as cells were placed on the stage (0 min). After 10 minutes the normal media was removed and replaced with media containing calcium (10 mM) and ionomycin (10 μM) for a 10 minutes incubation. The media was then removed and replaced with media lacking calcium. The Fura2 fluorescence was recorded at 10 seconds intervals with an imaging system composed of a filter wheel and a Basler A311F VGA camera connected to an Olympus IX71 inverted microscope. Data collection and the filter wheel were controlled by Automated *In Cyte2* software. Calcium concentration was measured by interpolation from a standard curve generated by calibration buffer kit #2 (Molecular Probes) and Fura2/K5-salt. Background fluorescence was corrected, and the intensity of the fluorescence (F340/F380) was monitored in 30–40 cells.

### 2.13 | Plasmid manipulation

The mammalian expression construct pcDNA3.1-hygro-PARP1-Myc-6His was a kind gift from Dr Saraswati Sukumar (Johns Hopkins University School of Medicine). Site-directed mutagenesis was done by following the protocol of the In-Fusion HD Cloning Kit (Clontech Laboratories). Table 1 lists the primers used in the amplification reactions, for generating potential ATR phosphorylation-deficient mutations of PARP1, for adding a FLAG tag to the C-terminal of PARP1, and for deleting the extra codons from the C-terminal of the original construct that do not belong to PARP cDNA.

### 2.14 | FLAG-tagged protein purification

Human HEK293T cells, mammalian expression plasmids pcDNA3.1-FLAG-ATR and pcDNA3.1-FLAG-PARP1 were used for recombinant protein purifications. Briefly, HEK293T cells were seeded overnight ahead of plasmid transfection. After 48 hours transfection the cells were harvested and lysed in ice-cold cellular lysis buffer (50 mM HEPES-KOH, pH7.4, 150 mM NaCl, 1 mM EDTA, 1% Triton X-100, 10% glycerol, and 1 × protease and phosphatase inhibitor cocktail) for 20 minutes at 4°C on a rotator. The cell lysate was clarified by centrifugation at 20 000 *g* for 10 minutes at 4°C. FLAG M2 magnetic beads were added to each tube of cell lysate, rotated for 4 hours at 4°C, and then collected with a magnetic rack. The collected beads were washed twice with buffer A (50 mM HEPES-KOH, pH7.4, 150 mM NaCl, 10% glycerol, 1 mM EDTA, and 0.05% NP-40), a high salt wash (50 mM HEPES-KOH, pH7.4, 1 M NaCl, 1 mM EDTA, and 0.05% NP-40) for 2 minutes on a rotator, and a final two washes with buffer A. Recombinant protein was eluted from the beads with 0.1 mL of elution buffer (50 mM Tris-HCl, pH 7.5, 150 mM NaCl, 10% glycerol, 100 μg/mL FLAG peptide, and 1 mM DTT) for 4 hours at 4°C on a rotator. The purity of the isolated proteins was determined by SDS-PAGE followed by Coomassie blue gel staining; their concentrations were determined by Coomassie blue binding using standard curves of serial dilutions of BSA. The eluted proteins were saved as aliquots that were snap frozen in liquid nitrogen and stored at −80°C.

### 2.15 | In vitro PARylation

The in vitro PARylation assay was done similarly as described by others.^71,72^ Briefly, PARP1 (20 nM) (Thermo Fisher Scientific or Flag-tag purified) and/or ATR (40 nM) (from our FLAG-tagged protein purification) were incubated at 4°C in a PARylation reaction buffer (100 mM Tris-HCl pH7.8, 10 mM MgCl_2_, 1 mM DTT, 25 μg/mL activated DNA oligo, and 200 μM NAD^+^) in a total volume of 40 μL in 1.5 mL micro-centrifuge tubes. The reaction was started by adding 200 μM ^32^P-NAD^+^ and increasing the temperature to 30°C for a 30 minutes incubation, then stopped by adding 2X SDS loading buffer with heating at 60°C for 5 minutes.

### 2.16 | In vitro phosphorylation

Cell extracts were prepared from HEK293T cells that were treated with 10 μM ionomycin for 2 hours followed by cell lysis for 10 minutes on ice in IP buffer (50 mM Tris-HCl, pH 7.8, 150 mm NaCl, 0.5% Nonidet P-40, 1 mM sodium fluoride, 10% glycerol, and 1 × protease and phosphatase inhibitor cocktail). Clarified supernatants were subjected to immunoprecipitation with anti-ATR antibody (Bethyl A300–137A) and protein A/G-agarose. The beads were washed three times with cell lysis buffer followed by two washes with kinase buffer (20 mm HEPES-KOH, pH 7.5, 50 mM NaCl, 10 mM MgCl_2_, 1 mM dithiothreitol, and 10 mm MnCl_2_). Finally, the immunoprecipitant was resuspended in 25 μl of kinase buffer containing 10 μCi of [γ−^32^P]ATP, 100 μM cold ATP, and 1 μg recombinant PARP1 protein substrate. The kinase reaction was conducted at 30°C for 20 minutes and stopped by the addition of 2X SDS-gel loading buffer. Proteins were separated by SDS-polyacrylamide gel electrophoresis and transferred to PVDF membrane. Immunoprecipitated ATR and recombinant PARP1 were confirmed by western blotting. Radiolabeled proteins were visualized and quantitated on a PhosphorImager (FUJI).

### 2.17 | Statistics and reproducibility

All data used for statistical analysis were from at least three repeats. Unpaired two-tailed *t* tests were performed to compare differences between two groups. One-way ANOVA was used to determine differences among the means of three or more groups. An unpaired two-tailed *t* test was performed to confirm where significant differences occurred within groups. A *P*-value <.05 was considered significant and is indicated by “*” in the figures.

## 3 | RESULTS

### 3.1 | ATR suppresses Ca^2+^ overload-induced necrosis following calcium ionophore treatment

**FIGURE 1:**
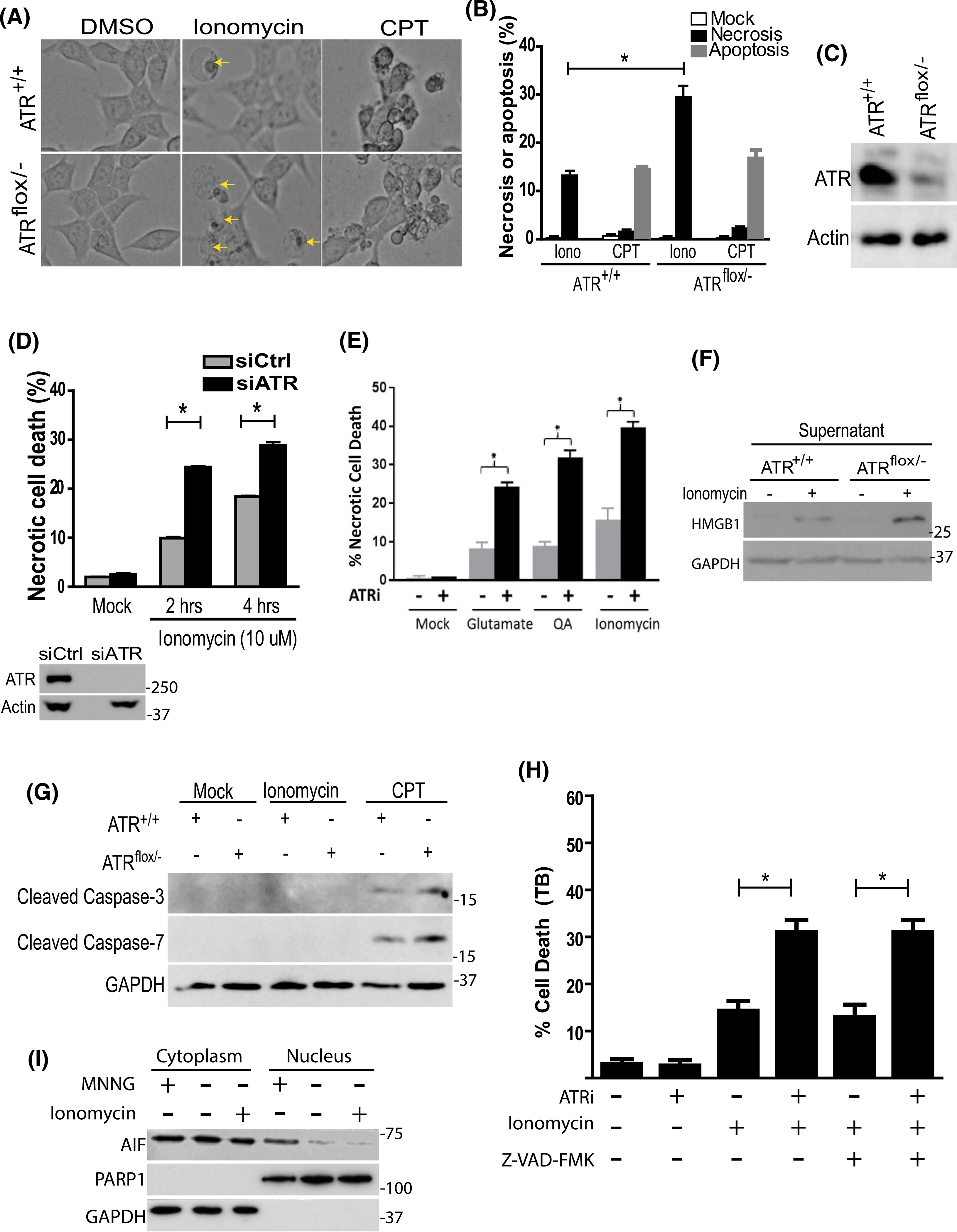
ATR inhibits intracellular Ca^2+^-induced necrosis. A, HCT-116 ATR^+/+^ and ATR^flox/−^ cells were treated with either ionomycin (10 μM, 4 h) or the apoptosis inducer camptothecin (CPT) (10 μM, 24 h) followed by phase-contrast imaging (20X). Necrosis, characterized by cellular swelling and nuclear shrinkage (yellow arrows), was exclusively induced by ionomycin treatment. B, Quantitation of the morphologic analysis of the three experiments as shown in (A). C, WB analysis of ATR levels in ATR^+/+^ and ATR^flox/−^ cells. D, Human A549 cells were transfected with siRNA specific for ATR followed by ionomycin treatment as indicated, then the percentage of cell death was measured via the trypan blue exclusion assay. E, Human SH-SY5Y neuronal cells were pre-treated with vehicle control or 80 nM of ATR inhibitor VE-822 (ATRi) for 30 min, then treated with glutamate (Glu, 100 μM), quinolinic acid (QA, 1 mM), or ionomycin (Iono, 5 μM) for 4 h. Cells were harvested and stained with trypan blue for necrotic cell counting. F, HCT-116 ATR^+/+^ and ATR^flox/−^ cells were treated with ionomycin for 4 h. HMGB1 released into the media was assessed by WB using the amount of GAPDH in the whole cell extract as loading control. G, WB analysis of the cleavage of caspase-3 and caspase-7 in ATR^+/+^ and ATR^flox/−^ HCT-116 cells treated with either ionomycin or CPT as in (B). H, HCT-116 ATR^+/+^ cells were pre-incubated with apoptosis inhibitor Z-VAD-FMK (10 μM, 1 h) and/or ATR kinase inhibitor VE-822 (10 μM, 1 h) followed by mock or ionomycin treatment (10 μM, 4 h). The percentage of necrotic cell death was measured using the trypan blue exclusion assay. I, Cells were treated with ionomycin (10 μM, 4 h) or MNNG (250 μM, x h; positive control), followed by fractionation and WB analysis. Quantitative data are shown is in Mean ± SD of triplicated experiments

Under physiological conditions, cells maintain a low cytosolic free-Ca^2+^ concentration of ~100 nM relative to the ~1.2 mM extracellular concentration. An increase of cytosolic Ca^2+^ to 200–400 nM may induce apoptosis.^73^ In contrast, a spike to a higher concentration of cytosolic Ca^2+^ due to uncontrolled influx across the plasma membrane may trigger necrosis.^33,74^ We initially explored ATR’s role in Ca^2+^ overload-induced necrosis after treating ATR-deficient cells (ATR^flox/−^) and the parental wild-type HCT-116 cells (ATR^+/+^) with ionomycin (Figure 1A–C). This Ca^2+^ ionophore dramatically raises intracellular levels of Ca^2+^ (Figure S1A).^74^ Morphological analysis of the cells indicated that ~30% of the ATR^flox/−^ cells underwent necrosis, characterized by cell swelling and plasma membrane rupture (Figure 1A,B), which was over two-fold higher than ionomycin-treated ATR^+/+^ cells which displayed ~13% necrotic death; untreated ATR^+/+^ cells exhibited little or no necrosis. Treatment of the cells with camptothecin, a topoisomerase I inhibitor, was employed as a control showing significant induction of typical apoptotic cells characterized by cell shrinkage and formation of apoptotic bodies (Figure 1A). A similar higher level of necrotic cell death was observed using the standard trypan blue exclusion assay in cells with siRNA knockdown of ATR (Figure 1D), and in multiple cell lines including primary human fibroblasts (Figure S1B,C).

We then assessed the effects of ATR kinase inhibition on necrosis with a mimicking cell system more relevant to brain ischemia. In this case, SH-SY5Y neuronal cells were treated with glutamate (Glut), quinolinic acid (QA), or ionomycin to induce an intracellular Ca^2+^ spike in the cells with or without ATR kinase inhibition. Glutamate and quinolinic acid are two known physiological and pathological stimuli that bind to glutamate receptors such as the NMDA receptor of neural cells to trigger an influx of extracellular calcium into these cells. As shown in Figure 1E, the remarkable similarity and consistency of the effect of ATR’s inhibition on necrotic cell death were observed in SH-SY5Y cells among these stimuli. The inhibition of ATR kinase significantly increased necrotic cell death in each of these conditions, suggesting the relevance of the effects to ischemic conditions. This implies that ATR may play a general role in suppressing calcium overload-induced necrosis.

Under our experimental condition, calcium influx into cells induced only necrotic cell death, as demonstrated by morphological analysis (Figure 1A,B) and the membrane breakage-associated release of HMGB1 (Figure 1F). No traceable amount of apoptotic cell death was be detected by morphological analysis (Figure 1A,B) or the cleavage of caspases 3 or 7 (Figure 1G) or PARP1 (Figure S1D). The involvement of ATR in the induced necrosis was validated further using the inhibitor VE-822 which demonstrated that inhibition of ATR elevated the necrosis by ~2 fold (Figure 1H). VE-822 is a potent ATR kinase-specific inhibitor that is currently in phase II clinical trials for cancer treatment and has been widely used in ATR studies. No changes in the level of necrosis were observed by treatment with the apoptotic cell death inhibitor Z-VAD-FMK (Figure 1H), further indicating that necrosis is the dominant form of cell death under our ionomycin treatment. Upon DNA damage AIF (Apoptosis-Inducing Factor) is released from mitochondria and migrates to the nucleus to induce apoptosis. Thus, we also examined the possible involvement of AIF in ionomycin-induced cell death. As shown in Figure 1I, little AIF was accumulated in nuclei after ionomycin treatment of cells relative to the nuclear accumulation of AIF after treatment with the DNA damaging agent N-methyl-N′-nitro-N-nitrosoguanidine (MNNG). These data indicate little or no activation of AIF by ionomycin treatment.

**FIGURE 2:**
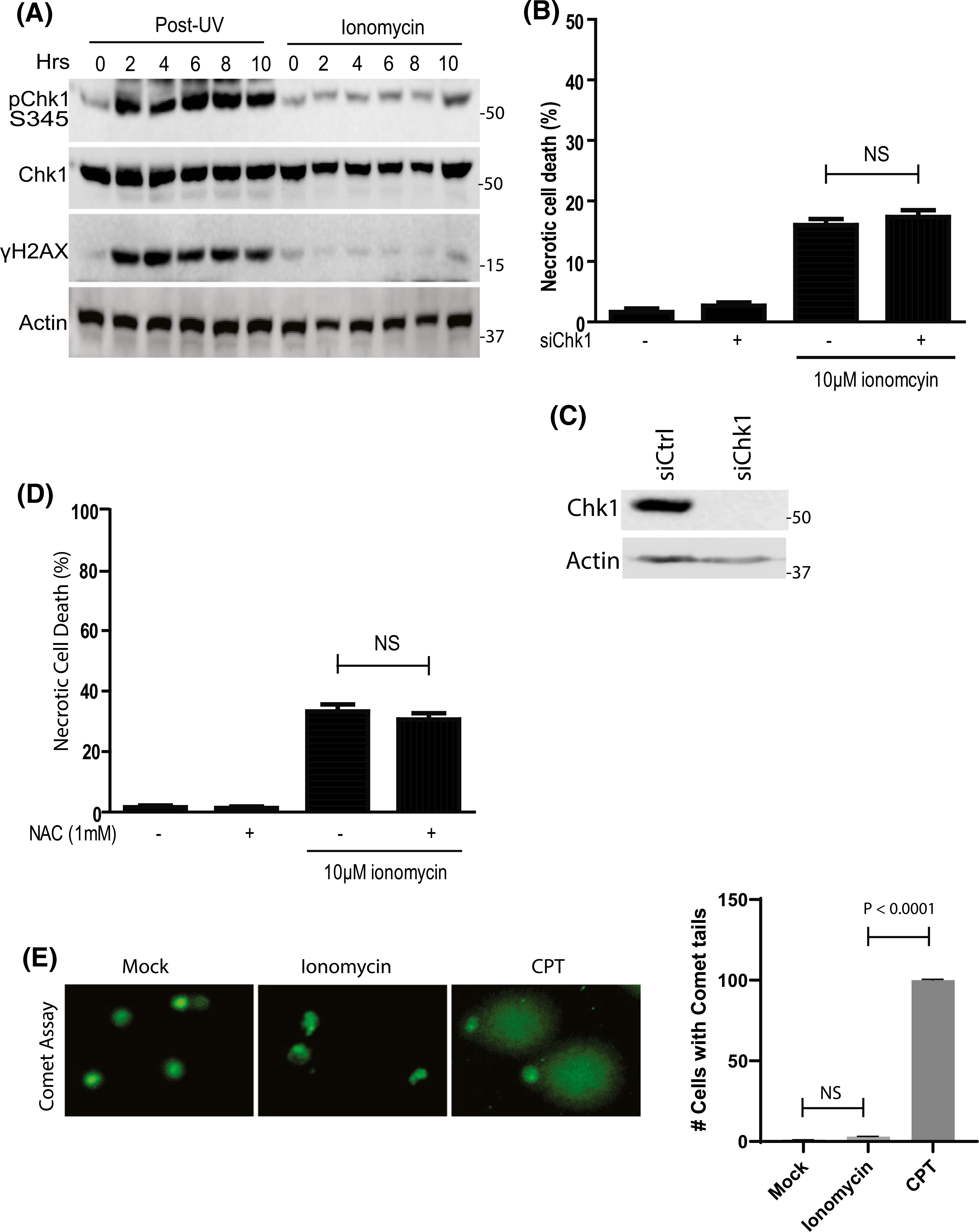
ATR kinase is not activated toward Chk1 phosphorylation during Ca^2+^ overload-induced necrosis. A, A549 cells were treated with 20 J/m^2^ UV-C and allowed to recover or with 10 μM ionomycin for indicated times. WB assessed the kinase activity of ATR toward its DDR substrates Chk1 and H2aX. B, Chk1 was silenced by siRNA knockdown. At 48 h post-transfection, cells were treated with 10 μM ionomycin for 4 h, and the percent necrotic cells determined by the trypan blue exclusion method (mean ± SD of triplicated experiments). C, WB analysis of Chk1 knockdown efficiency. D, A549 cells were pre-incubated with mock or 1 mM N-acetyl-L-cysteine (NAC) for 1 h, then 10 μM ionomycin was added and incubation continued for another 4 h. Mean ± SD of three replicates on the percentage of necrotic cells is shown. E, Cells were treated with ionomycin (20 μM), CPT (20 μM) or mock-treated and then subjected to an alkaline comet assay analysis. Quantitation was performed based on over 100 cells each sample

Interestingly, the canonical ATR DNA damage signaling cascade through Chk1 and H2AX was not observed in cells treated with ionomycin (Figure 2A,B), even with a prolonged 24 hours treatment. These results suggest that the activity of ATR under Ca^2+^ overload was not related to a DNA damage response, at least not for the first 24-hour period. This contrasts with the traditional view that ATR functions only as a guardian of the genome. In addition, efficient knockdown of Chk1, the major mediator of ATR-anchored DNA damage signaling, did not affect Ca^2+^ overload-induced cell death (Figure 2B,C). Preincubation of cells with the antioxidant N-acetyl-L-cysteine (NAC) had little or no effect on necrotic cell death (Figure 2D), suggesting that an increase in reactive oxygen species (ROS) is not critical for the Ca^2+^ overload-induced necrotic cell death during the early hours of treatment. Furthermore, the ionomycin-treated cells also were assessed by the comet assay, showing little or no DNA breaks produced in cells (Figure 2E). In contrast, CPT treatment, as a positive control, generated a large amount of DNA damage. Together, all these results support a role of ATR in a novel non-DNA damage response to Ca^2+^ overload.

### 3.2 | Kinase activity of ATR is involved in the inhibition of necrotic cell death

**FIGURE 3:**
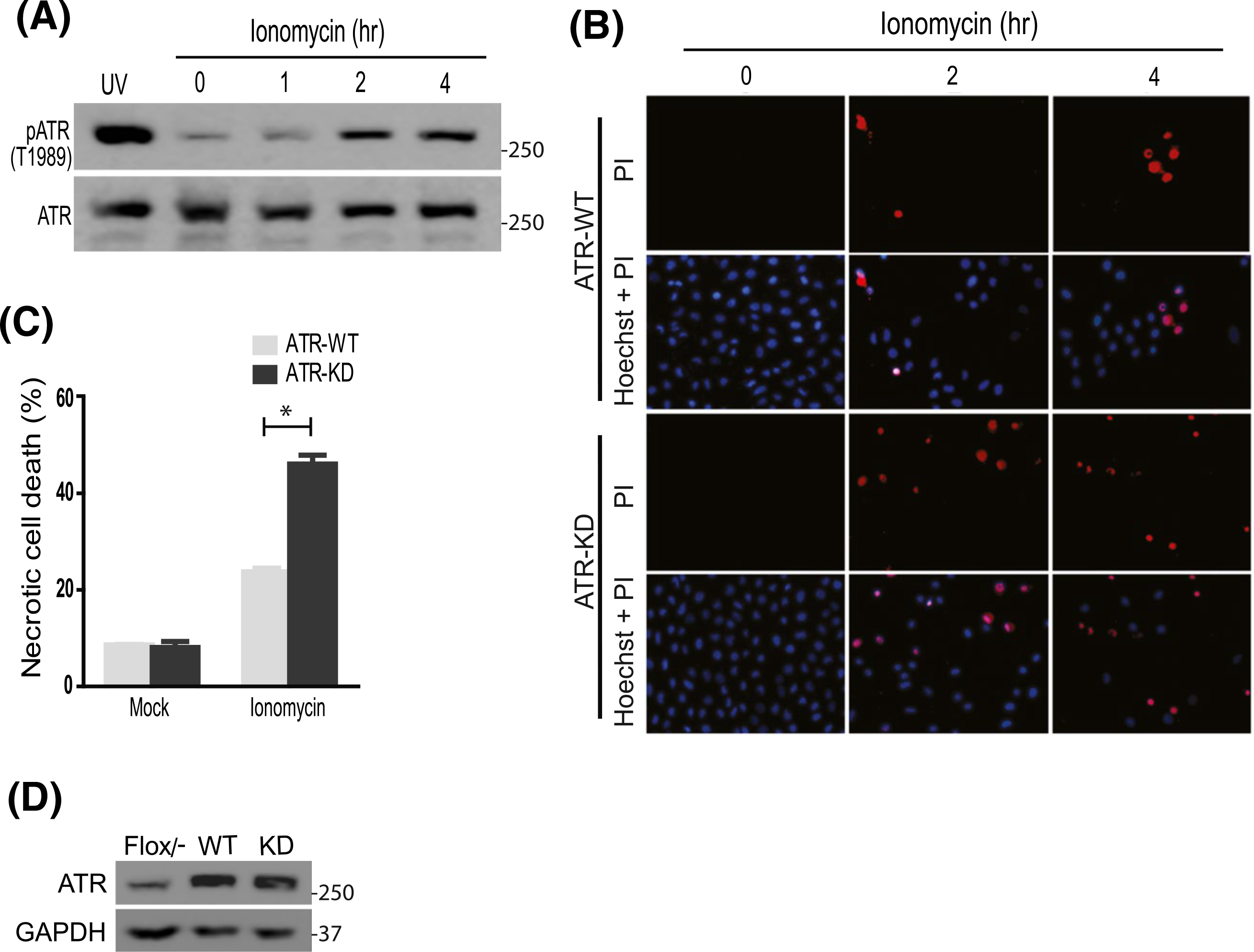
The anti-necrotic function of ATR is dependent on its kinase activity. A, WB analysis of ATR phosphorylation on T1989 in A549 cells after UV-C irradiation (40 J/m^2^, 2 h recovery) or 10 μM ionomycin for the times indicated. B and C, ATR-WT and ATR-KD plasmids were transfected into ATR^flox/−^ cells and, at 48 h post transfection (~70% efficiency), treated with ionomycin (10 μM, 4 h). The percentage of necrotic cell death was assessed using the PI and Hoechst staining assay followed by fluorescent microscopy (B) and quantified as the mean ± SD of three independent experiments (C). D, WB analysis of ATR-WT and ATR-KD protein expression by constructs in ATR-deficient cells (Flox/−) using anti-ATR antibody

Our data in Figure 1H implies a role of ATR kinase activity in suppressing Ca^2+^ overload-induced necrosis. Autophosphorylation of ATR on threonine 1989^75,76^ is a marker for and a switch of ATR to kinase-active status. To assess if ATR kinase is active after the treatment we examined the level of ATR phosphorylated on T1989 [pATR (T1989)] as an indicator of kinase activity and observed a significant upregulation after ionomycin treatment (Figure 3A). We then transfected ATR^flox/−^ cells with wild-type (WT) or kinase dead (KD) ATR expression constructs and assessed the level of necrotic cell death by propidium iodine staining (Figure 3B–D). A ~2-fold increase in necrotic cell death was observed (Figure 3B,C) in cells expressing ATR-KD, which is similar to the degree of necrotic death with ATR kinase inhibition (Figure 1H), ATR deficiency (ATR^flox/−^, Figure 1B,C), and ATR knockdown (Figure 1D).

### 3.3 | PARP1 is a causative factor of the Ca^2+^ overload-induced necrosis and is inhibited by ATR kinase

**FIGURE 4:**
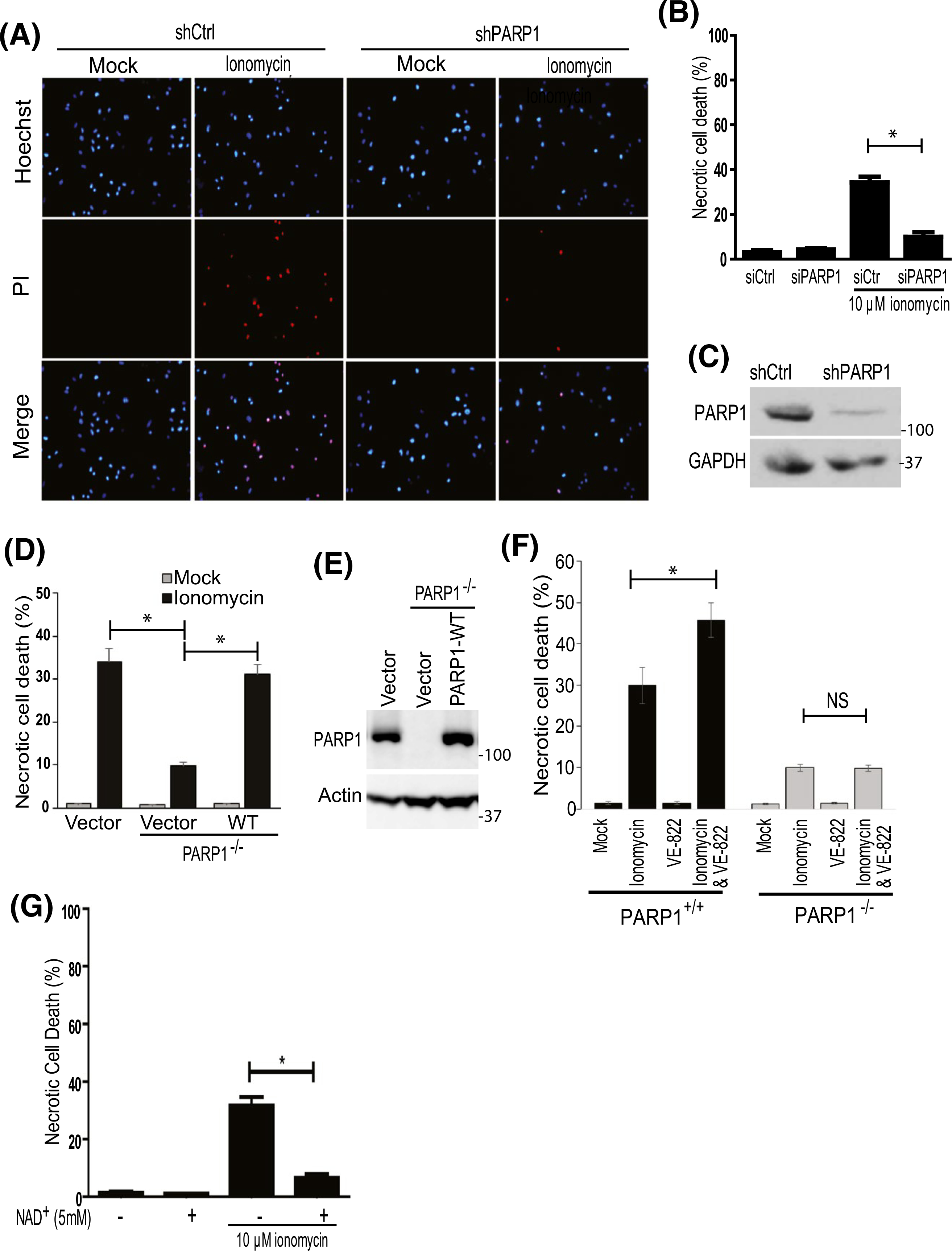
PARP1 is the mediator of the anti-necrotic role of ATR kinase. A, Stable A549 shCtrl and shPARP1 cells were mock or ionomycin treated for 2 h, followed by PI and Hoechst staining. B, Quantitation of the mean ± SD of three independent experiments as shown in (A). C, WB analysis of PARP1 expression in A549 shCtrl and shPARP1 cells. D and E, U2OS-WT, U2OS PARP1 knockout (PARP^−/−^), and the PARP1 knockout cells stably transfected with empty vector (Vector) or wild-type PARP1 (WT) were treated with 10 μM ionomycin or vehicle control (Mock), followed by trypan blue staining. The mean ± SD data on necrotic cells from three biological repeats and the level of PARP1 expression are shown in (D) and (E), respectively. F, U2OS (PARP^+/+^) and U2OS PARP1 knockout (PARP1^−/−)^ cells were preincubated with ATR inhibitor VE-822 (80 nM, 30 min), followed by 10 μM ionomycin for 2 h followed by trypan blue staining. G, A549 cells were supplemented with or without β-NAD^+^ (10 mM) for 1 h, followed by treatment with ionomycin (10 μM, 2 h), and the mean ± SD from three independent experiments on cell viability via the LDH assay are shown

Ca^2+^ overload leads to hyper-activation of PARP1 and excessive consumption of NAD^+^ in cells, which is a well-known cause of necrotic cell death by causing ATP depletion.^36,46,49,51^ We hypothesized that PARP1 is a mediator of ATR’s role in moderating Ca^2+^ overload-induced necrosis. shRNA knockdown (Figure 4A–C and Figure S2), knockout (Figure 4D,E), or inhibition (Figure S2) of PARP1 in cells reduced Ca^2+^ overload-induced necrosis following ionomycin treatment. Importantly, the reduced necrotic cell death in PARP1-null cells (PARP1^−/−^) was restored by expressing exogenous PARP1 (Figure 4D,E and Figure S2C), consolidating that the Ca^2+^ overload-induced necrosis is dependent on PARP1 and PARylation. Inhibition of ATR kinase elevated the level of necrotic cell death in PARP1^+/+^ cells but has no such effect in the PARP1^−/−^ cells (Figure 4F), suggesting that PARP1 is, indeed, the major mediator of ATR’s anti-necrotic role in the Ca^2+^ overload-induced necrotic responses. In addition, supplementing cells with β-NAD^+^ reduced necrosis (Figure 4G), further supporting the role of PARP1 as the causative factor of Ca^2+^ overload-induced necrosis. These findings also suggest an underlying mechanism requiring PARP1-mediated PARylation in this necrotic cell death.

### 3.4 | Ca^2+^ overload induces PARP1-mediated hyperPARylation which is inhibited by ATR kinase

**FIGURE 5:**
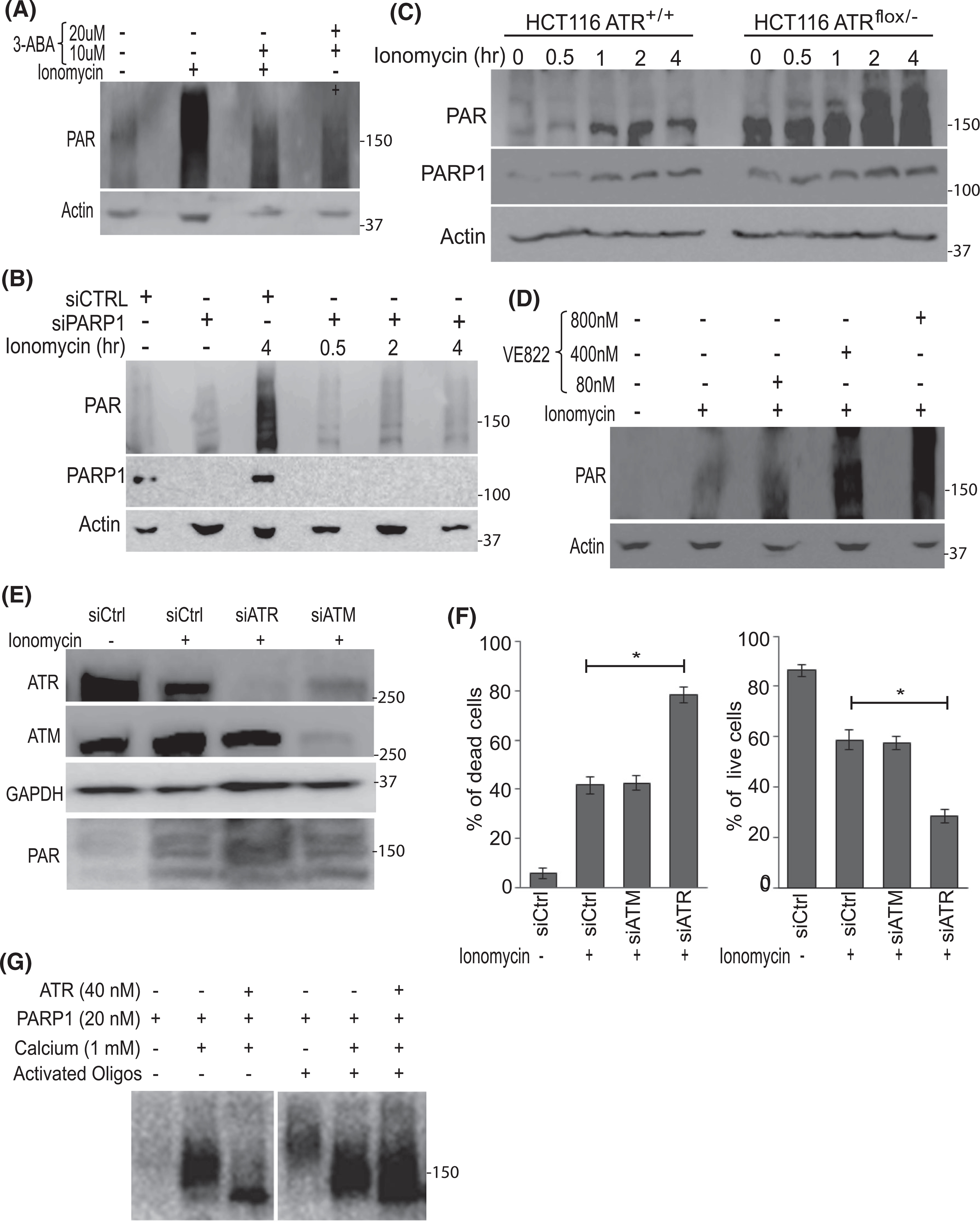
ATR kinase inhibits intracellular Ca^2+^-induced PARylation by PARP1. A, A549 cells were treated with ionomycin (10 μM, 4 h) with or without PARP1 inhibitor 3-ABA at the indicated concentrations. The level of PAR polymers in the cells are detected by WB analysis using anti-PAR antibody. B, A549 cells were transfected with siRNA specific for PARP1 (siPARP1) or a scrambled siRNA (siCTRL) for 48 h, followed by ionomycin (10 μM) or mock treatment for the indicated times. Cell lysates were analyzed by WB for PARylation using anti-PAR antibody. C, WB analysis of PAR polymers in HCT116 ATR^+/+^ and ATR^flox/−^ cells that were treated with ionomycin (10 μM) for the times indicated. D, WB analysis of PAR polymers in A549 cells treated with ionomycin (10 μM, 4 h) in the presence or absence of ATR kinase inhibitor VE-822 (10 μM, 1 h pre-incubation). E and F, A549 cells were transfected with siRNAs targeting ATR, ATM or a scrambled siRNA (siCtrl), and the efficiency of knockdown were confirmed with WB (E). Cells then were treated with 10 μM ionomycin for 4 h and the percentage of dead cells (left panel) and live cells (right panel) were assessed using the trypan blue exclusion and Cell Titer-Blue assays, respectively. The data are shown as mean ± SD of three repeats (F). G, The in vitro PARylation activity of PARP1, as affected by recombinant ATR protein or chemicals, was assessed using a PARylation assay in presence of ^32^P-NAD^+^. PARP1, which had incorporated ^32^P-NAD^+^, was separated on an 8% SDS-PAGE gel, transferred to PVDF membrane and the PARylated PARP1 was displayed by autoradiographic imaging

The above data identified an anti-necrotic role of ATR in Ca^2+^ overload-induced necrosis, which is through PARP1 depletion of NAD^+^. We further explored the mechanisms through which ATR may inhibit the PARP1-mediated necrotic activity in response to Ca^2+^ overload. We first determined the level of PARylation after Ca^2+^ overload. Ionomycin-induced Ca^2+^ overload robustly increased the level of PARylation (Figure 5A,B). Over 80% of the elevated PARylation was inhibited by PARP1 inhibitor 3-ABA or PARP1 knockdown (Figure 5A,B), suggesting that PARP1 is the enzyme responsible for most of the PARylation in response to Ca^2+^ overload. ATR deficiency further increased the Ca^2+^ overload-induced PARylation, particularly at the later time points (between 2 and 4 h) of ionomycin treatment (Figure 5C) when cells started to die (Figure S1B,C). In addition, in a dose-dependent manner, inhibition of ATR kinase increased Ca^2+^ overload-induced PARylation (Figure 5D).

Interestingly, siRNA knockdown of ATM, a similar DNA damage checkpoint kinase, did not elevate the level of PARylation as did the knockdown of ATR kinase (Figure 5E,F), suggesting that ATR’s responses to cellular Ca^2+^ overload is unique. Further studies demonstrate that Ca^2+^ stimulates PARP1-mediated in vitro PARylation activity (Figure 5G left) and that addition of purified ATR protein inhibited the extension of PARylation and PARs remain short. Interestingly, when short double-stranded oligonucleotides, which represent DNA double-strand breaks, were added to stimulate basal PARP1 activity as a positive control, the inhibitory effect of ATR on extension of PAR polymers, though still obvious, was over-shadowed by strand break-induced additional PARylation and extension (Figure 5G right). This suggests that PARP1 may be activated by Ca^2+^ overload and DNA damage in different ways, which is consistent with our observation that Ca^2+^ overload produced little DNA damage signaling during necrosis of cells. As such, our results indicate that ATR inhibits intracellular Ca^2+^ overload-induced PARylation.

### 3.5 | Ca^2+^ overload induces ATR-PARP1 binding, and subsequent phosphorylation of PARP1 by ATR to suppress PARylation and necrotic cell death

**FIGURE 6:**
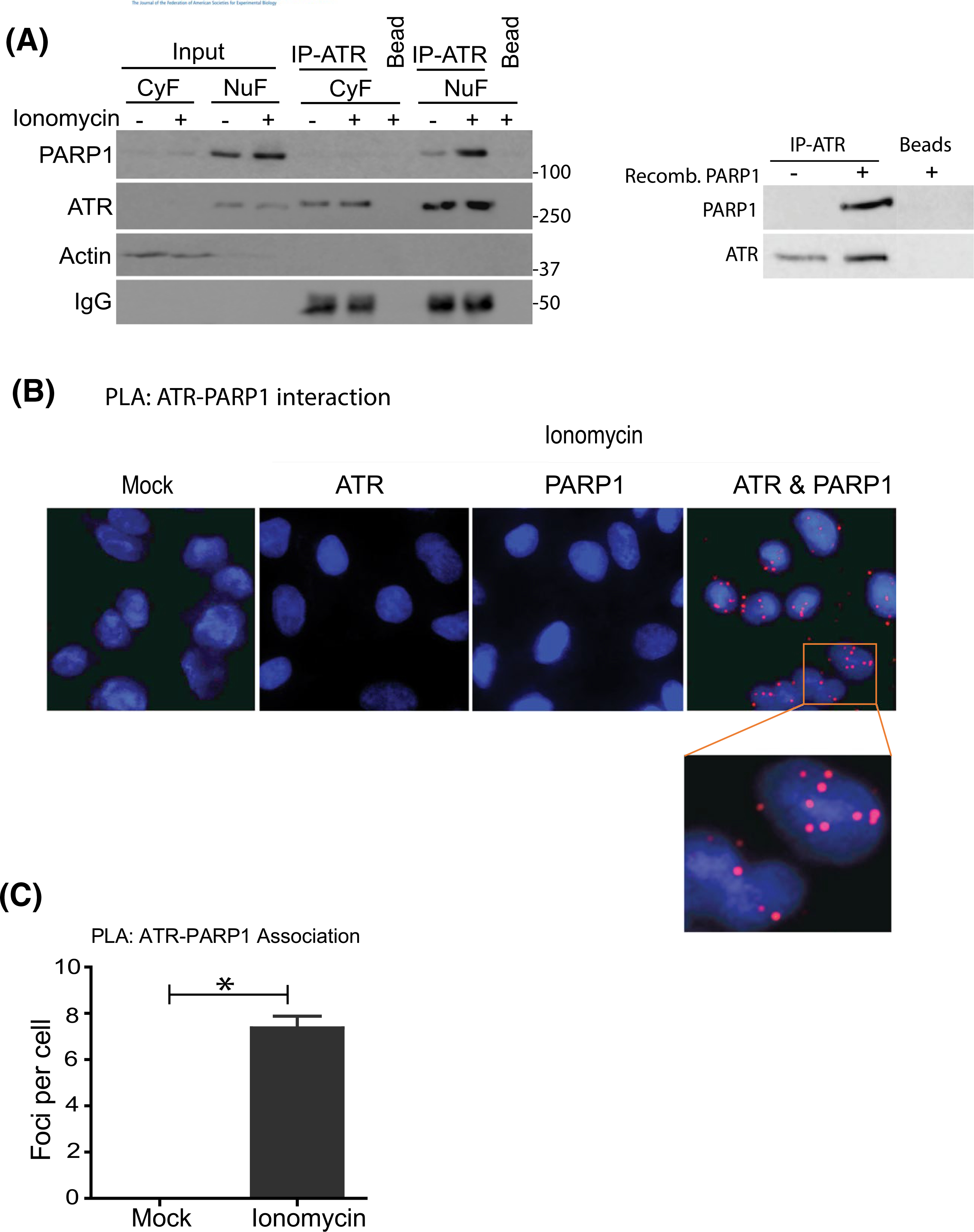
Ca^2+^ overload induces ATR-PARP1 complex formation. A, A549 cells were treated with ionomycin (10 μM) for 4 h before subcellular fractionation. ATR was immunoprecipitated (IPed) from both cytoplasmic and nuclear fractions (CyF and NuF, respectively), using anti-ATR antibody. The coimmunoprecipitation of PARP1 with ATR was analyzed by WB (left panel). IPed ATR was also subjected to a high salt wash to remove endogenous PARP1 and then was incubated with purified recombinant PARP1 in a binding buffer. After incubation the ATR was IPed again and the amount of bound PARP1 measured by western blot analysis (right panel). B and C, A549 cells were treated with ionomycin (10 μM, 2 h) followed by the proximity ligation assay (PLA) to detect ionomycin-induced ATR-PARP1 binding. Red dots indicate ATR-PARP1 complex formation under fluorescent microscopy (B). The quantitation of the mean ± SD of the foci in the PLA assay of 100 cells is shown in (C)

Having determined that ATR kinase inhibits PARP1-mediated PARylation to suppress Ca^2+^ overload-induced necrosis, we investigated the mechanisms by which ATR works through PARP1, possibly by phosphorylation of PARP1. We first examined possible ATR-PARP1 binding in different cell lines treated with ionomycin to induce necrosis. After mock or ionomycin treatment ATR was immunoprecipitated from nuclear lysates of A549 cells (Figure 6) or from whole cell lysates of HCT-116 cells (Figure S3). As shown in Figure 6A, the immunoprecipitated ATR pulled down significantly more nuclear PARP1 with than without Ca^2+^ overload (left) and this was confirmed by an *in vitro* binding assay showing ATR interaction with purified recombinant PARP1 (right). This complex formation was further confirmed by the Duolink proximity ligation assay (PLA) (Figure 6B,C); this is an effective complement to the immunoprecipitation assay because it assesses protein complex formation in cells after fixation. Given the fact that ATR prevented extension of PAR formation during PARylation (Figure 5), these results suggest that ATR may recognize and bind to PARP1 mono-PAR or a short polymer of PAR after Ca^2+^ overload.

**FIGURE 7:**
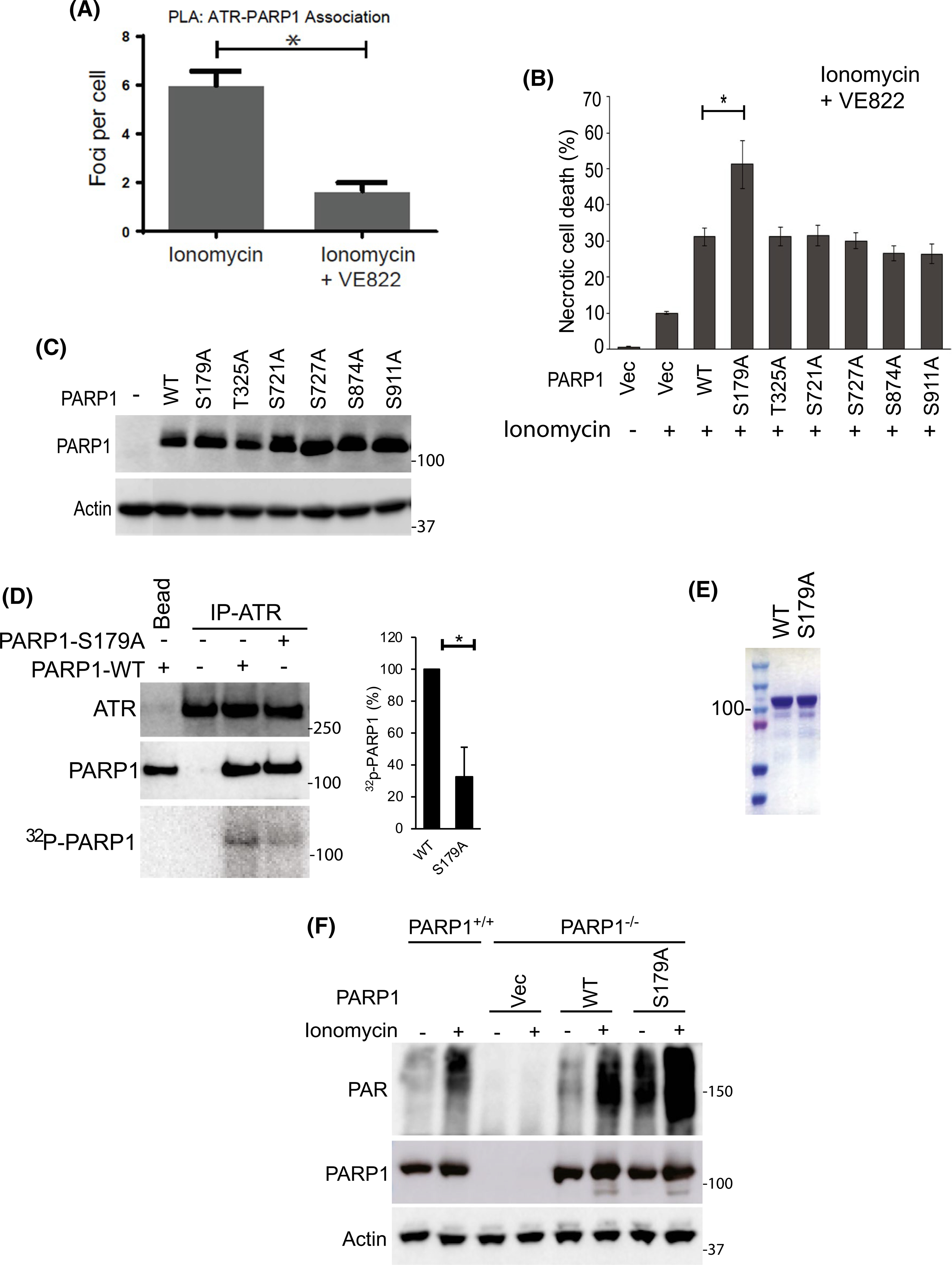
ATR phosphorylation of PARP1 at Ser^179^ inhibits PARylation and decreases Ca^2+^ overload-induced necrosis. A, A549 cells were treated with ionomycin (10 μM, 2 h) in the presence or absence of ATR kinase inhibitor VE822, followed by PLA to detect ionomycin-induced ATR-PARP1 interaction. Fluorescent foci (ATR-PARP1 interaction) per cell were quantified from three independent measurements. B, Mapping of the ATR phosphorylation site in PARP1 that is required for inhibition of Ca^2+^ overload-induced necrosis. The serines in all SQ/TQ sites in PARP1 predicted as phosphorylation candidates for ATR kinase were mutated to Ala. These mutants and the WT constructs were transfected into U2OS PARP1 knockout cells. Stable clones expressing these constructs were treated with ionomycin (10 μM, 2 h) and necrotic cell death assessed by trypan blue staining from three repeats. The first two lanes are the U2OS PARP1 knockout cells with PcDNA3.1 empty vector transfection (Vec). C, WB analysis determined that similar levels of PARP1 were expressed in each PARP1-containing cell line. D, In vitro phosphorylation of PARP1-WT and PARP1-S179A by immunoprecipitated ATR from ionomycin-treated (10 μM, 2 h) HEK293T cells. WB analysis demonstrated that equivalent levels of ATR and PARP1 were employed. The phosphorylation status of PARP1 was monitored by detecting the amount of ^32^P incorporated into the recombinant PARP1 proteins (bottom panel). E, Coomassie-stained SDS-PAGE gel showing a major single band both in purified PARP1-WT and PARP1-S179A lanes. F, The level of PARylation was assessed in the parental U2OS wild type (PARP1^+/+^) cells, and U2OS PARP1 knockout cells (PARP1^−/−^), cells with empty vector (Vec) or stably expressed PARP1^+/+^ (WT) or PARP1-S179A (S179A) proteins

We, therefore, hypothesized that this ATR-PARP1 complex formation may reflect ATR interacting with and then phosphorylating PARP1. Indeed, inhibition of ATR kinase activity by the specific inhibitor VE822 dramatically reduced ATR-PARP1 association as demonstrated by PLA assay (Figure 7A). We analyzed the PARP1 protein sequence (NCBI Ref Seq: NP_001609.2) and identified six potential SQ/TQ motifs as candidates for ATR phosphorylation^66,67,77^: serine 179, threonine 325, serine 721, serine 727, serine 874, and serine 911 of PARP1. We examined the effects of the potential phosphorylation on necrotic cell death by individually mutating each of these six amino acids to alanine (Figure 7B,C). Among the U2OS PARP1 knockout cells that stably express similar levels of WT or different PARP1 mutants (Figure 7C), PARP1-S179A is the only mutation that elevated necrotic cell death (Figure 7B). We then assessed the capability of immunoprecipitated ATR to phosphorylate PARP1 in an in vitro kinase assay, supplying purified PARP1-WT and PARP-S179A proteins from ionomycin-treated cells as substrates. ATR was able to add ^32^P to purified PARP-WT but much less ^32^P was added to PARP-S179A (Figure 7D), suggesting that S179 of PARP1 is indeed a site that is directly phosphorylated by ATR. It should be noted that the minimal amount of phosphorylation on PARP-S179A mutant may suggest the possibility that ATR also could phosphorylate other SQ/TQ sites of PARP1 though it is minor and might not be involved in necrosis regulation. The specificity of the phosphorylation of PARP1 by ATR was verified by pretreatment of immunoprecipitated ATR with ATR inhibitor (80 nM), which significantly reduced the in vitro PARP1 phosphorylation (Figure S4) (the remaining phosphorylation of PARP1 in the presence of ATRi is likely due to the incomplete inhibition by the inhibitor or the necrosis-independent phosphorylation or both). In addition, functionally, cellular PARP1-S179A showed a higher basal activity and Ca^+2^-induced hyperactivity of PARylation, particularly after the cells were treated with ionomycin to induce necrosis (Figure 7F). Interestingly, the background PAR level in the PARP1-S179A mutant cells (no ionomycin treatment) is significantly higher than in the WT cells. This is consistent with the result in Figure 5C where ATR deficient cells had a much higher background PAR level than WT cells (0 h ionomycin treatment), further supporting PARP1-S179’s essential role in PARP1 activity. These data suggest that the phosphorylation of S179 is necessary and sufficient for ATR inhibition of PARP1 PARylation activity.

## 4 | DISCUSSION

We report here that ATR suppresses Ca^2+^ overload-induced necrotic cell death, independent of DNA damage and AIF activity. The activation of ATR kinase activity is not related to DNA damage signaling, the well-accepted role of ATR. Rather, ATR kinase is activated in response to Ca^2+^ overload and phosphorylates PARP1 on serine 179 in the absence of detectable DNA damage signaling. The phosphorylation of PARP1 suppresses PARP1-mediated PARylation and the necrotic cell death induced by Ca^2+^ overload.

It is well known that Ca^2+^ overload-induced necrosis contributes to diseases such as heart failure, ischemic stroke, brain injury, and immune diseases, typically involving early necrosis and delayed apoptosis.^2–13,15,17,78,79^ Necrosis is responsible for the initial or early cell death during ischemia.^2,11,17^ The extent and degree of PARP1 hyperactivation has been demonstrated to be a cause of necrotic cell death by consuming NAD^+^ leading to ATP depletion.^36–39^ It is worth mentioning that necrosis as described here and parthanatos are both driven by hyper-activation of PARP, but occur via two different mechanisms^18^: Necrosis is characterized by NAD^+^/ATP depletion,^9,17–19^ while parthanatos involves DNA damage, AIF activation and inhibition of glycolysis rather than NAD^+^/ATP depletion.^19,80–82^ Our results successfully recaptured the fundamental mechanisms that cells use to initiate necrosis during Ca^2+^-overload (Figures 4 and 5). However, little is known about how PARP1 activity is regulated. In the current study, our results show that, in contrast to the phosphorylation by Chk2 to activate PARP1 in the DDR,^83^ ATR phosphorylation of PARP1 in response to Ca^2+^ overload inhibits its PARylation activity. Consistently, PARP1 appeared to be activated differently by DNA damage and Ca^2+^ overload (Figure 5G); in the former case, PARP1 is activated (but not hyper-activated) to promote the DDR, while in the latter PARP1 is hyper-activated to induce necrosis which is inhibited by ATR. Specifically, the current study showed that phosphorylation of PARP1 at serine 179 by ATR is necessary for ATR suppression of Ca^2+^-overload-induced necrosis (Figure 7D). Our finding that PARP1 with a point mutation at this phosphorylation site (Ser179Ala) showed elevated PARylation activity, with and without Ca^2+^-overload (Figure 7F), strongly supports this proposed mechanism. It is possible that the phosphorylation of PARP1 may have similar roles in other stress responses, since phosphorylation of this same site has been identified previously by mass spectrometry in DNA damage responses.^77^

ATR is well known for its regulatory role in DDR, as a DNA damage checkpoint kinase, to signal the presence of DNA damage and to regulate cell cycle checkpoints,^57,75,84,85^ DNA repair^70,86–93^ and apoptotic cell death. ATR suppresses apoptosis through the Chk1 kinase pathway during apoptotic cell death.^94^ ATR also can localize to mitochondria via direct interaction with tBid^69^ and suppress tBid-mediated apoptosis independent of its kinase activity in response to DNA damage by ultraviolet irradiation and other agents. In addition, ATR promotes nucleotide excision repair (NER) via physical interaction with the repair protein xeroderma pigmentosum group A (XPA).^92^ Our work here indicates that ATR also plays a role in non-DNA damage stress responses by a novel mechanism. As shown in Figure 2A, γH2AX and phospho-Chk1-S345, as the important indicators of ATR kinase activity in the DDR, were not detected in ionomycin-treated cells.

Our results indicate a Ca^2+^ overload induction of autophosphorylation of ATR on Thr1989, an indicator of kinase-active ATR (Figure 3A). While how Ca^2+^ overload activates the ATR-PARP1 anti-necrotic pathway remains to be determined, our analysis indicates that PARP1 may contain a potential EF hand-like Ca^2+^ binding motif to which calcium may bind to activate PARP1. Moreover, our results reveal that an increase in ATR-PARP1 complex formation (Figure 6) inhibits PARylation to prevent extension of PAR polymers (Figure 5) in response to Ca^2+^-overload. These data suggest that ATR may recognize partially PARylated PARP1. We speculate that the initial auto-PARylation of PARP1 recruits and then activates ATR to phosphorylate PARP1, which in turn suppresses further PARP1 activity. In addition, our results show that ionomycin treatment induced nuclear condensation (Figure 1A) and, interestingly, this Ca^2+^-dependent nuclear condensation is PARP1 dependent also (Figure S2C). It has been reported that high calcium levels induce over-condensation of DNA-polycation complexes in vitro.^95–98^ Such over-condensation would induce replication and transcription stresses which might activate ATR toward PARP1.^99,100^ However, it is also possible that other unknown Ca^2+^-dependent proteins or kinases might directly activate the ATR-ATRIP complex via phosphorylation in a DNA damage-independent manner. Further investigation into the role of PARP1 PARylation and the length of the PAR polymers is needed but is out of scope of the current study.

Interestingly, a PARP1-ATR interaction was reported previously in cells treated with low doses, but not high doses, of the DNA damage agent methyl methanesulfonate (MMS). ATR is a substrate for PARylation by PARP1 in this type of DNA damage.^72^ Inhibition of PARP1 dissociates the ATR-PARP1 complex formed with low dose of MMS.^72^ PARP1 also is a sensor of intracellular DNA damage and replication stress and PARylation is the first wave of stress signaling for recruiting other DDR factors.^101^ Clearly, our results indicate that cells respond to DNA damage and Ca^2+^ overload by different mechanisms in terms of the roles of ATR and PARP1. Finally, this report has not specifically addressed how Ca^2+^ overload activates ATR, but our findings present an interesting insight deserving of further investigation in the future.

## Supplementary Material

Supplementary Info

## Figures and Tables

**TABLE 1:** Primers that were used for site-directed mutagenesis

PARP1-S179A-F: 5′-GCGGCTCAGCTCAAGGGCTTCAGCCTCCTTGCTAC-3′
PARP1-S179A-R: 5′-CTTGAGCTGAGCCGCACTGTACTCGGGCCGGAAAC-3′
PARP1-T325A-F: 5′-GTCAAGGCACAGACACCCAAC-3′
PARP1-T325A-R: 5′-TGTCTGTGCCTTGACCATACACTTG-3′
PARP1-T368A-F: 5′-GCCGCGCCTCCGCCCTCCACAGCCTCGGCTCCTGCT-3′
PARP1-T368A-R: 5′-GGGCGGAGGCGCGGCCGCCACGGAGGCGCTGGTT-3′
PARP1-S721A-F: 5′-GCGGTGGCTCAGGGCAGCAGCGACTCTCAGATCCTGGAT-3′
PARP1-S721A-R: 5′-GCCCTGAGCCACCGCCTGCTGGACCTCACTGAGGATGGA-3′
PARP-1-S727A-F: 5′-CGACGCTCAGATCCTGGATCT-3′
PARP-1-S727A-R: 5′-AGGATCTGAGCGTCGCTGCT-3′
PARP1-S874A-F: 5′-GGATCCTGGCTCAGGGTCTT-3′
PARP1-S874A-R: 5′-CCTGAGCCAGGATCCCAGCAAA-3′
PARP1-S911A-F: 5′-ACTGCCATACGGCTCAGGGAGA-3′
PARP1-S911A-R: 5′-GAGCCGTATGGCAGTAGTTGGC-3′
PARP1-FLAG-F: 5′-GACTACAAAGACGATGACGACAAGTAACTCGAGTCTAGAGGGCCCGTT-3′
PARP1-FLAG-R: 5′-GACTACAAAGACGATGACGACAAGTAACTCGAGTCTAGAGGGCCCGTT-3′
PARP1-DelC-F: 5′-CTGTGGTAACTCGAGTCTAGAGGGCCCGTT-3′
PARP1-DelC-R: 5′-CTCGAGTTACCACAGGGAGGTCTTAAAATTGAATTTCAG-3′

## Abbreviations:

AIF: apoptosis inducing factor
ATM: ataxia telangiectasia mutated
ATP: adenosine triphosphate
ATR: ataxia telangiectasia and Rad3 related
BSA: bovine serum albumin
DAPI: 4′, 6-diamidino-2-phenylindole
DMEM: Dulbecco’s modified Eagle’s medium
DDR: DNA damage responses
DMEM: Dulbecco’s modified Eagle’s medium
ER: endoplasmic reticulum
FBS: fetal bovine serum
Glut: glutamate
HMGB1: high mobility group box 1
IP: immunoprecipitation
IPed: immunoprecipitated
KD: kinase dead
MNNG: N-methyl-N′-nitro-N-nitrosoguanidine
NAC: N-acetyl-L-cysteine
NAD^+^: nicotinamide adenine dinucleotide
PBS: phosphate-buffered saline
PAR: poly (ADP-ribose)
PARP1: Poly [ADP-ribose] polymerase 1
PARylation: poly ADP-ribosylation
PI: propidium iodide
PLA: proximity ligation assay
QA: quinolinic acid
ROS: reactive oxygen species
U2OS: human osteosarcoma cell line
VE-822: ATR kinase inhibitor
WB: western blot
WT: wild type
Z-VAD-FMK: apoptotic cell death inhibiton
