# Supplementary material for: ATR prevents Ca2+ overload-induced necrotic cell death through phosphorylation-mediated inactivation of PARP1 without DNA damage signaling
Source: FASEB J. Author manuscript; Available in PMC 2021 Jul 9. (PMC8252533; doi:10.1096/fj.202001636RRR)
Supplement: Supplementary Info [file NIHMS1713375-supplement-Supplementary_Info.pdf]

Supplementary Figure 1

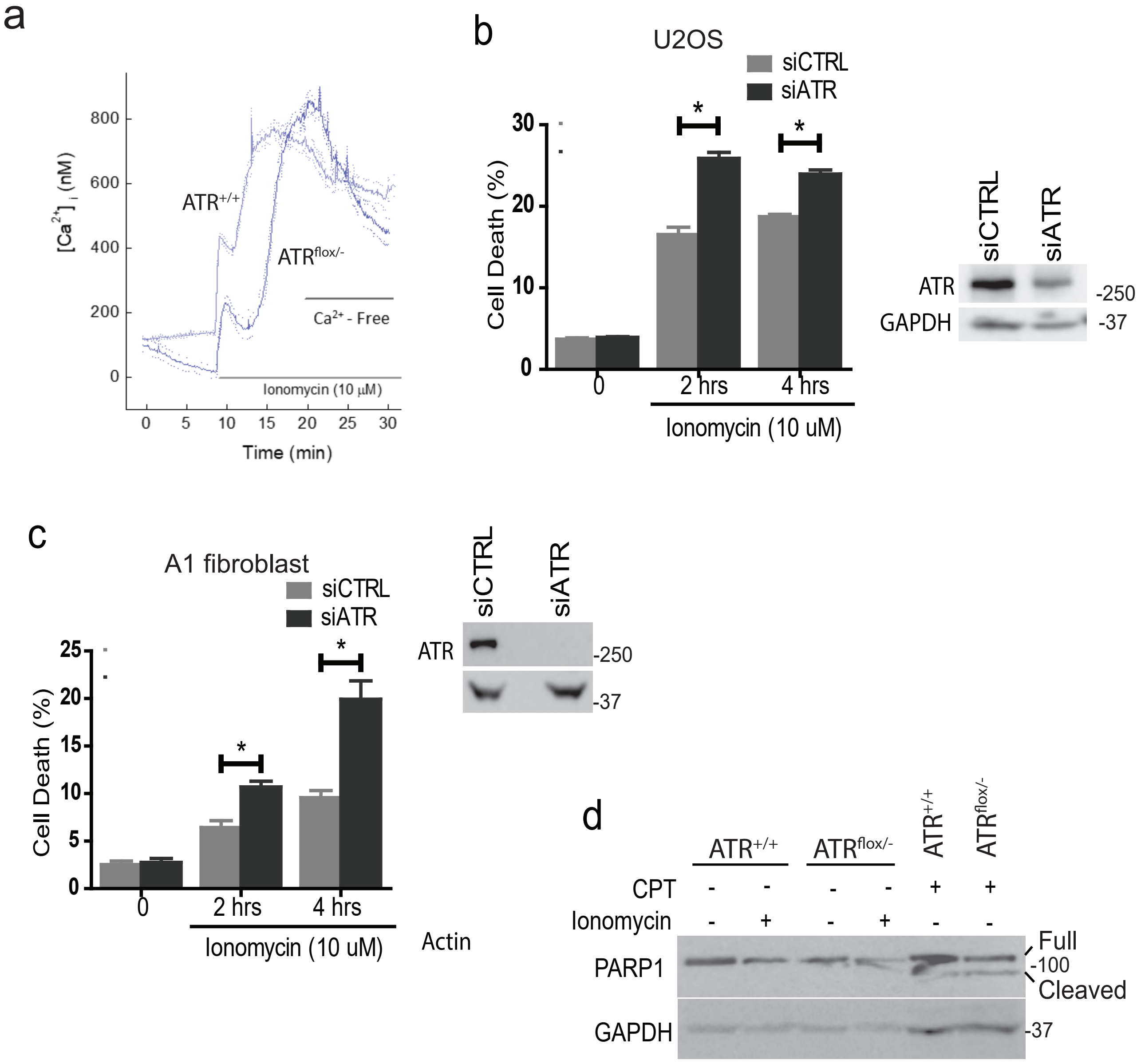

Supplementary Figure 1. ATR plays a role in inhibiting Ca^{2+} overload-induced necrotic cell death. (a) Intracellular Ca^{2+} levels spike in HCT-116 $ATR^{+/+}$ and $ATR^{flox/-}$ cells treated with ionomycin. Briefly, cells in Ca^{2+}-containing media were incubated with the intracellular calcium indicator Fura-2 before ionomycin treatment. Intracellular Ca^{2+} concentration was measured for 10 minutes before ionomycin was added, then for 10 min after ionomycin addition, followed by another 10 minutes of measurement in Ca^{+2}-free media containing ionomycin. **(b), (c)** Human U2OS cells or A1 fibroblast cells (passage 18) were transfected with ATR-specific siRNA for 48 hr followed by ionomycin treatment as indicated; cell death was measured employing the trypan blue exclusion assay. The lower panels display a WB analysis of ATR knockdown in these cells. **(d)** WB analysis of the cleavage of PARP1 in $ATR^{+/+}$ and $ATR^{flox/-}$ cells treated with either ionomycin or CPT as in Fig. 1g.

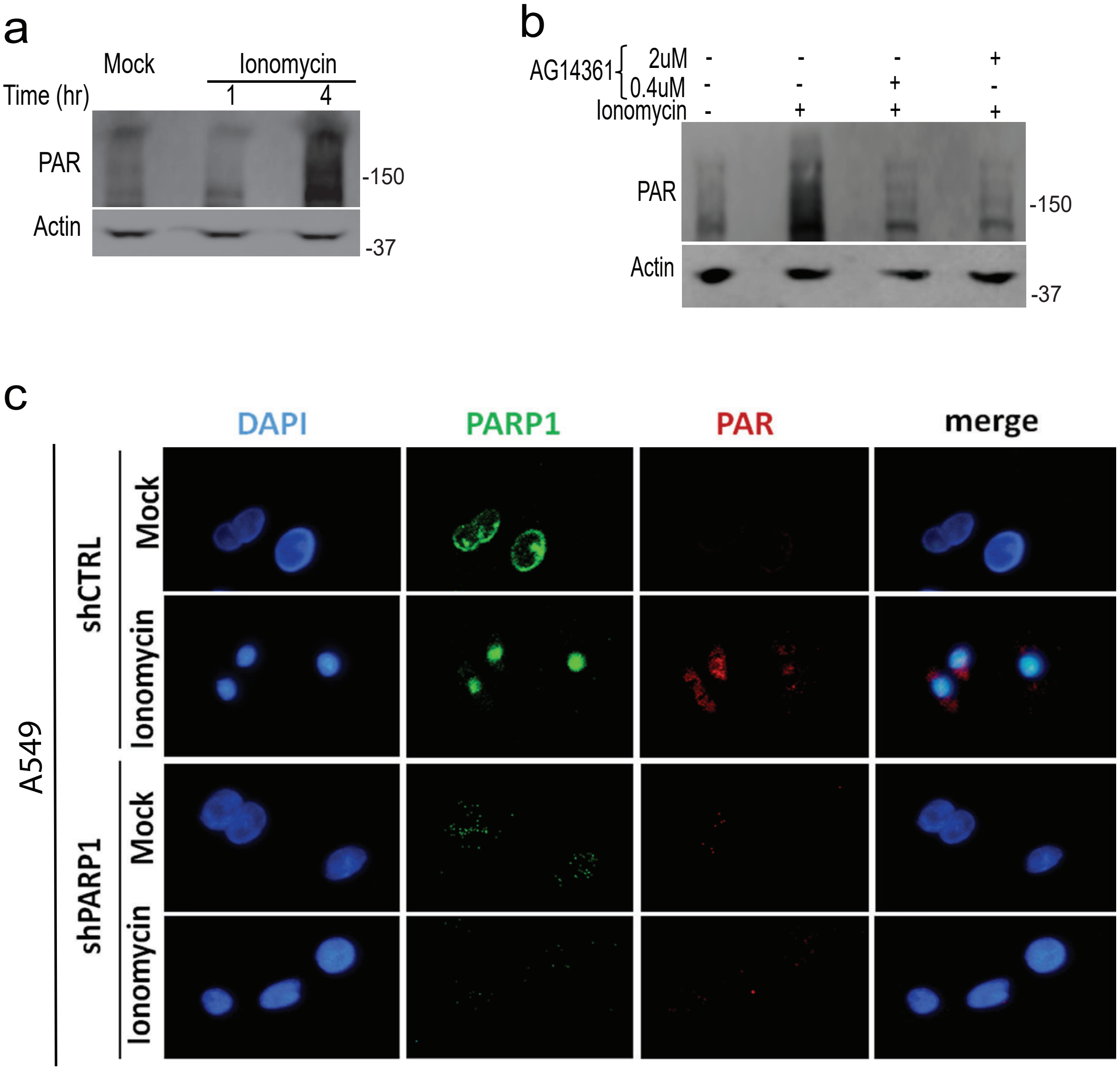

Supplementary Figure 2. Validation of PARylation. **(a)** A549 cells were treated with ionomycin (10 µM, 1 and 4 hours). Formation of PAR polymers are detected by WB analysis using anti-PAR antibody. **(b)** A549 cells were treated with 0, 0.2 or 0.4 µM PARP1 inhibitor AG14361 for 1 hr, then ionomycin (10 µM) was added for an additional 2 hrs, followed by WB analysis of formation of PAR polymers using anti-PAR antibody. **(c)** Human A549 shPARP1 and shCTRL cells were mock or ionomycin (10 µM) treated for 2 hours, followed by immunofluorescence using DAPI staining and the indicated antibodies.

Supplementary Figure 3

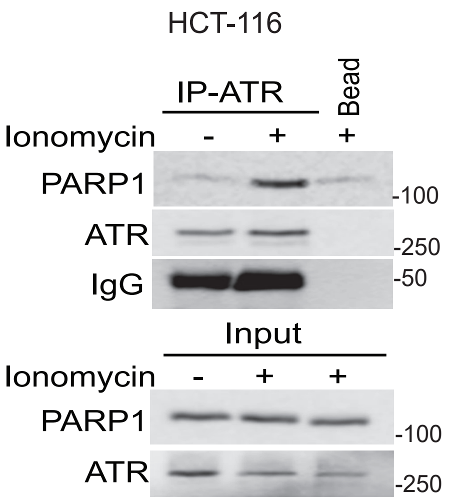

Supplementary Figure 3. ATR-PARP1 complex formation in response to intracellular Ca^{2+} stress is observed in HCT-116 cells. Human HCT116 cells were treated with ionomycin (10 µM, 4 hrs) followed by fractionation and immunoprecipitation of ATR as described in Fig. 6a.

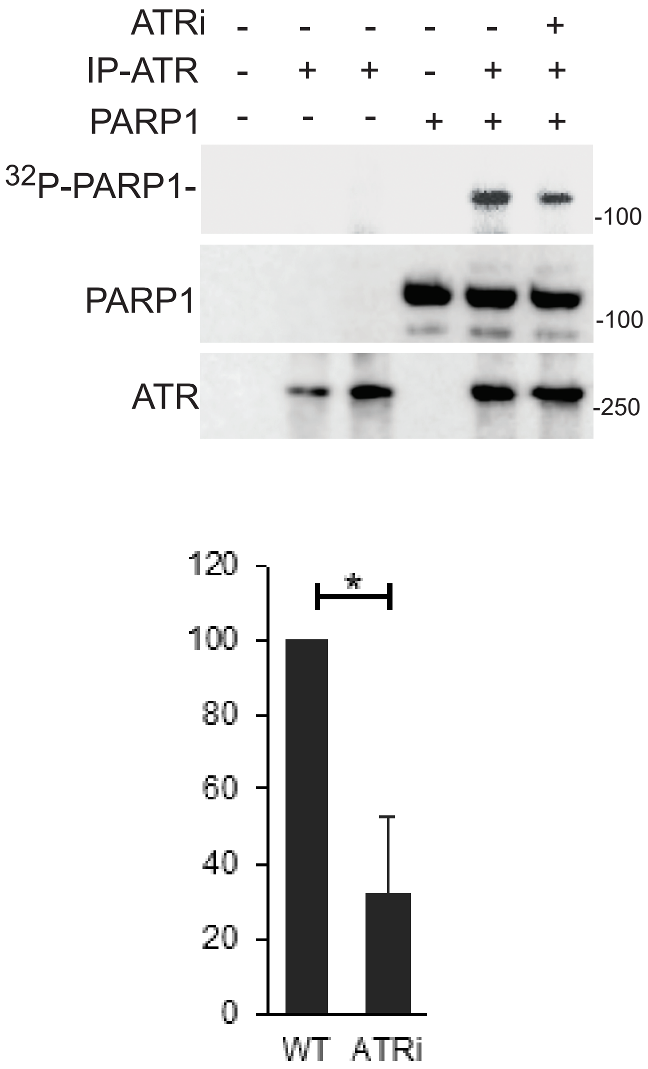

Supplementary Figure 4. ***In vitro*** **phosphorylation of recombinant PARP1-WT by immunoprecipitated ATR from ionomycin-treated (10 µM, 2 hrs) A549 cells.** The *in vitro* assay of PARP1 phosphorylation by IPed ATR with [γ-^{32}P]ATP was conducted to show that ATR can directly phosphorylate PARP1, while addition of ATR kinase inhibitor VE-822 (ATRi, 80 nM) significantly reduced the phosphorylation. The status of phosphorylation on PARP1 was shown at top by detecting the amount of ^{32}P incorporated into the recombinant PARP1 protein.
